# Interplay of Anatomy and Surgical Approach: A Comparative Review of Neurovascular Risk in Lateral and Oblique Lumbar Interbody Fusion

**DOI:** 10.7759/cureus.102022

**Published:** 2026-01-21

**Authors:** Ali Hamide, Maryam Babar, Aymen Arain, Masab A Mansoor, Jordan Bendavid, Razi Rashid

**Affiliations:** 1 Medical School, Edward Via College of Osteopathic Medicine, Monroe, USA; 2 Internal Medicine, Edward Via College of Osteopathic Medicine, Monroe, USA; 3 Neurology, University of Texas Medical Branch, Houston, USA

**Keywords:** intraoperative neuromonitoring, lateral lumbar interbody fusion, lumbar plexus, neurovascular complications, oblique lumbar interbody fusion, psoas major, retroperitoneal approach

## Abstract

Degenerative conditions of the lumbar spine, such as disc herniation, spinal stenosis, and degenerative spondylolisthesis, represent a significant global health burden, frequently leading to chronic pain, neurological deficits, and diminished quality of life. Traditional open surgical approaches for lumbar interbody fusion, while effective, often involve extensive soft tissue dissection, which can lead to considerable blood loss, prolonged recovery times, and muscle damage. In response to these challenges, minimally invasive surgical (MIS) techniques have rapidly evolved, aiming to achieve comparable clinical outcomes with reduced iatrogenic tissue injury. Among the prominent MIS strategies for lumbar interbody fusion, two approaches have gained significant traction: lateral lumbar interbody fusion (LLIF), which encompasses techniques such as the direct lateral transpsoas interbody fusion (LTIF), and oblique lumbar interbody fusion (OLIF). Both techniques offer distinct advantages by providing access to the lumbar disc space from a corridor largely anterior to the posterior spinal elements, thereby minimizing disruption to the paraspinal musculature and bony structures. However, these benefits come with unique anatomical challenges. The success and safety of both LLIF/LTIF and OLIF are intrinsically linked to a precise understanding and careful navigation of the retroperitoneal space, particularly concerning the intricate lumbar plexus and associated major vascular structures. The proximity of vital nerves and vessels to the surgical corridors presents a significant risk of iatrogenic injury, which can lead to severe neurological deficits, vascular complications, and compromise patient outcomes. Furthermore, the inherent anatomical variability of the lumbar plexus and vascular structures among individuals adds another layer of complexity to these approaches, necessitating meticulous preoperative planning and intraoperative vigilance. This comparative review aims to synthesize the current literature on the distinct anatomical considerations and associated neurovascular risks encountered during lateral lumbar interbody fusion (LLIF/LTIF) and oblique lumbar interbody fusion (OLIF) approaches. By elucidating the specific anatomical challenges of each technique and the strategies employed to mitigate complications, we seek to provide a comprehensive overview for clinicians and researchers involved in the field of minimally invasive spinal surgery.

## Introduction and background

Understanding the lumbar plexus: general anatomy and clinical significance

The lumbar plexus (LP) is a web of nerve fibers that consists of the anterior (ventral rami) of the L1-L4 spinal nerve roots and descends through the posterior abdominal wall in the retroperitoneal space to reach the lower limb [1, 2]. It is there that the nerves innervate their target structures, providing sensation, motor function, or both to their respective anatomical landmarks. The posterior (dorsal rami) of the L1-L4 spinal nerve roots travel posteriorly to the deep muscles of the back and their overlying skin [3]. The objective of this review is to provide a direct anatomical comparison between the LLIF and OLIF approaches, specifically regarding neurovascular risk and surgical corridors. 

One of the main anatomical relations to the LP is the psoas major muscle, which often contains part of the LP embedded within its dense fibrous fascia [4]. The psoas major muscle consists of an anterior and posterior portion that is separated by this fascia. The anterior portion of the muscle begins at the anterolateral surface of the intervertebral disc and vertebral bodies of T12 to L5, and the posterior portion of the muscle often originates more laterally and deep towards the transverse processes of the T12 to L5 vertebrae [3]. It is between these two muscular bellies within the fascia that the LP is formed. The psoas muscle itself also receives small branches from the femoral nerve [5].

The LP consists of six major nerves. In order from superior to inferior, the nerves are as follows: iliohypogastric nerve, ilioinguinal nerve, genitofemoral nerve, lateral femoral cutaneous nerve, femoral nerve, and obturator nerve. The first nerve in the plexus, the iliohypogastric nerve, arises from the T12-L1 spinal levels [6]. It passes posterior to the lower pole of the kidney and anteriorly to the quadratus lumborum muscle [6]. It then pierces the posterior part of the transversus abdominis muscle just superior to the iliac crest, where it provides the internal oblique and transversus abdominis muscles with motor innervation [3]. It also provides sensation to the skin over the lower anterior abdominal wall, the suprapubic region, and the superior lateral gluteal region [6].

The Ilioinguinal nerve consists of the L1 ventral rami and travels just parallel and inferior to the iliohypogastric nerve [3]. The ilioinguinal nerve delivers sensation to the overlying skin of the anterior superior and medial areas of the thigh [6]. In males, its terminal branches develop into the anterior scrotal nerve, which carries sensory information from the base of the penis and the upper scrotum [7]. In females, these branches become the anterior labial nerves, responsible for sensation in the skin of the mons pubis and labia majora [7]. The nerve also sends motor fibers to the internal oblique and transversus abdominis muscles, assisting their function alongside the iliohypogastric nerve [6].

The genitofemoral nerve contains contributions from both the L1 and L2 ventral spinal nerve roots [7]. The genitofemoral nerve descends inferiorly, piercing the psoas major muscle, and then courses through the retroperitoneal space, traveling along the anterior aspect of the psoas major muscle itself [7]. As it continues downward, the nerve separates into the femoral and genital branches. Upon reaching the inguinal region, the genitofemoral nerve enters the deep inguinal ring and passes through the inguinal canal. In males, the genital portion innervates the cremaster muscle, where it provides motor input for elevating the testicles as well as sensory innervation to the scrotum [6]. In females, it provides sensation to the mons pubis and the labia majora [7]. The femoral branch provides sensory innervation to the anterior, superior area of the thigh in both genders [7].

The lateral femoral cutaneous nerve arises from the ventral rami of the L2 and L3 spinal nerves. Its primary and sole function is to provide sensory innervation to the skin covering a large portion of the lateral thigh, extending from just below the hip down toward the knee. Unlike many other nerves in the lumbar plexus, it does not carry any motor fibers [3]. The fifth and largest nerve of the LP is the femoral nerve, which gets its contributions from the L2, L3, and L4 ventral spinal nerve roots. The femoral nerve passes deep to the middle 1/3 of the inguinal ligament just lateral to the femoral vessels in the femoral triangle, where it divides into the cutaneous and muscular branches [7]. The cutaneous branch is responsible for sensation in the anterior compartment of the thigh, and the muscular branch innervates the hip flexors: pectineus, iliacus, sartorius, and knee extensors: quadriceps, rectus femoris, vastus medialis, vastus lateralis, and vastus intermedius [6].

The most inferior nerve in the LP is the obturator nerve, which also arises from the L2, L3, and L4 spinal nerve roots [7]. The obturator nerve traverses the pelvis to enter the thigh via the obturator foramen, where it then divides; its anterior branch travels inferiorly between the adductor longus and adductor brevis muscles, and its posterior branch descends between the adductor brevis and adductor magnus muscles. It provides both motor and sensory supply to the muscles of the medial thigh compartment [7].

A wide range of pathological processes, such as iatrogenic surgical complications, neoplasms, traumatic injury (motor vehicle accidents, stab wounds), infectious agents, and inflammatory or microvascular disorders, can compromise the integrity of the lumbar plexus, giving rise to a condition known as lumbosacral plexopathy. The resulting clinical manifestations depend on the precise segment of the plexus involved. LP injuries commonly present with pain in the lower extremities, occurring in 20-50% of cases [8]. Patients with lumbar plexopathy experience weakness of the involved musculature in roughly 55% of cases [8].

The symptoms of nerve damage can be isolated to the affected nerve in the LP based on clinical presentation and the specific motor and sensory deficits that a patient may be experiencing. In the case of a genitofemoral nerve lesion, ongoing pain or discomfort localized near the superior inguinal ligament, extending toward the anterior thigh and genital region, often occurs in conjunction with hypoesthesia or hyperesthesia in the external genitals [9]. In the case of Iatrogenic injury to the plexus, especially during lateral lumbar interbody fusions, the L4-L5 levels have been shown to have the greatest involvement of post-surgical complications due to being located inside the operative zone in 3.7-66.7% of cases [10]. Damage to the contributions to the femoral nerve or obturator nerve may lead to a lack of sensation in the anterior medial thigh and weakness during hip flexion, knee extension, and thigh adduction [11]. Overall, injuries to the nerves of the LP can lead to a decrease in the quality of life of a patient, as they often lead to chronic pain and disability. Early recognition and proper surgical precautions are essential to minimize damage and limit long-term complications of LP injuries.

It is imperative to recognize the anatomical variations that exist between patients and how these variations may impact the LP and its surrounding anatomy, especially during surgery. The ilioinguinal nerve demonstrates a range of anatomical differences, including variations in its origin, branching pattern, and anatomical course, and the nerve may be absent in 12% of individuals [12]. The main observed variations of the lateral femoral cutaneous nerve were the presence of an accessory lateral femoral cutaneous nerve that had a prevalence of approximately 3% and an aberrant branching of the nerve from the femoral nerve in roughly 8% of cadavers studied in a sample of 47 cases [13].

Given the wide range of anatomical variations within the LP, careful consideration and thorough knowledge of these differences are essential to minimize surgical risks and optimize patient outcomes during interventions taking place near this complex network of nerves. This review first defines the surgical corridors for LLIF and OLIF, examines the neurovascular structures that constrain these windows, and finally provides a decision-making framework for selecting the appropriate approach based on patient-specific anatomy.

## Review

Search strategy and selection criteria

**Figure 1:**
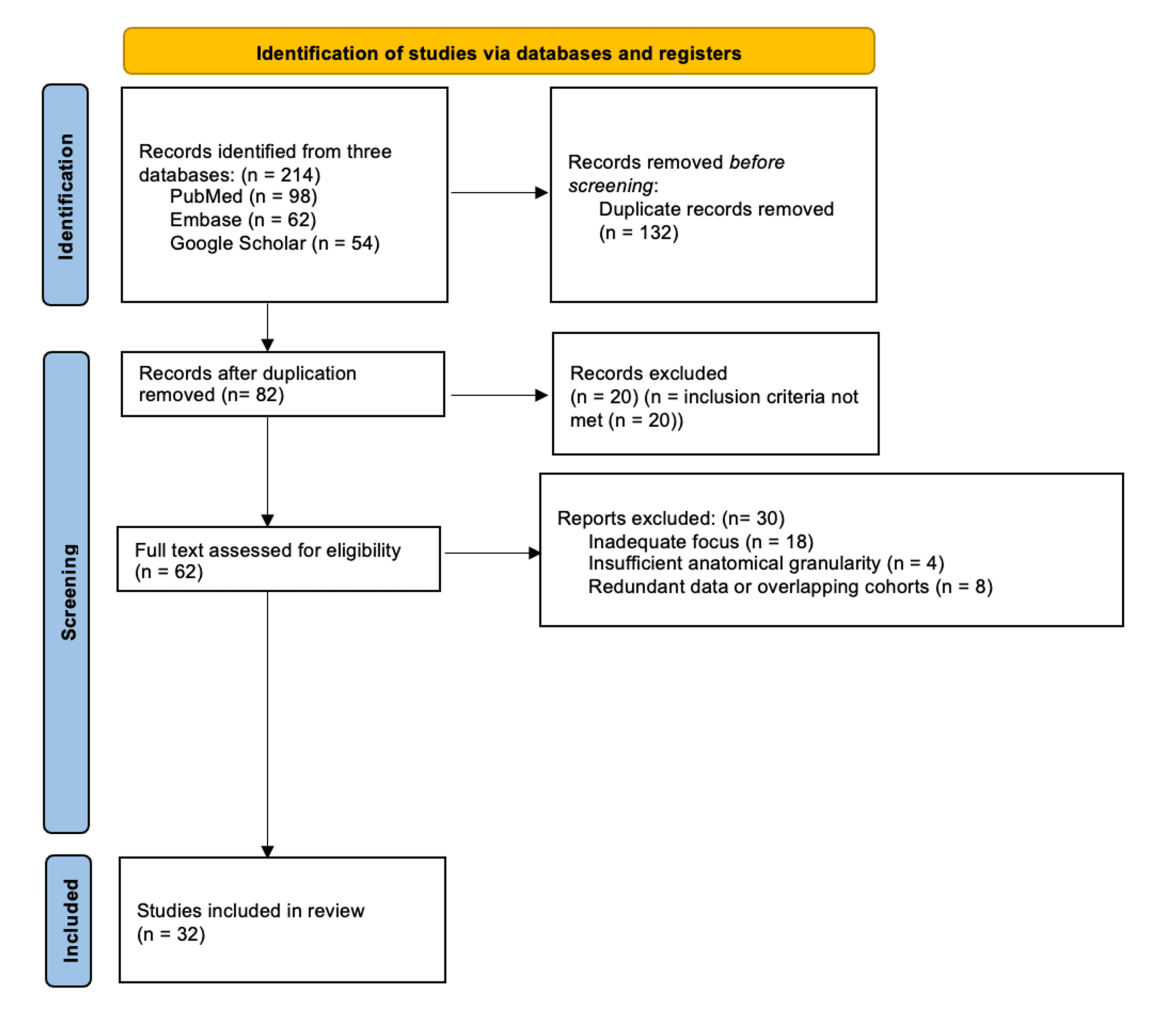
PRISMA Diagram PRISMA: Preferred Reporting Items for Systematic Reviews and Meta-Analyses

Data for this review were identified by searches of PubMed, Embase, and Google Scholar for articles published from inception to December 2024. The literature search and study selection process were conducted in accordance with Preferred Reporting Items for Systematic Reviews and Meta-Analyses (PRISMA) guidelines and are summarized in the PRISMA flow diagram (Figure 1). Search terms included combinations of the following keywords: "lateral lumbar interbody fusion," "LLIF," "oblique lumbar interbody fusion," "OLIF," "lumbar plexus anatomy," "surgical corridors," "transpsoas approach," and "pre-psoas approach." The selection process focused on high-quality anatomical studies, large-scale clinical series, and systematic reviews. Priority was given to articles that provided: 1. Direct comparative data between the transpsoas and pre-psoas corridors. 2. Detailed mapping of neurovascular structures (e.g., femoral nerve, sympathetic chain, and iliac vessels) relative to surgical landmarks. 3. Clinical outcomes specifically reporting approach-related neurological or vascular complications. 4. Only peer-reviewed articles published in English were included. Reference lists of identified articles were also manually searched for additional relevant studies.

Inclusion Criteria

The inclusion criteria were: 1. Peer-reviewed clinical trials, prospective and retrospective cohort studies, systematic reviews, and cadaveric anatomical studies. 2. Studies specifically evaluating Lateral Lumbar Interbody Fusion (LLIF/transpsoas) and/or Oblique Lumbar Interbody Fusion (OLIF/pre-psoas). 3. Articles providing detailed mapping of the lumbar plexus, the great vessels, or the sympathetic chain in relation to the surgical corridors. 4. Studies reporting on approach-related complications, including neurological deficits (e.g., sensory/motor changes), vascular injury, or psoas muscle morbidity. 5. Articles published in English from database inception to December 2025.

Exclusion Criteria

The exclusion criteria were: 1. Studies focusing exclusively on traditional posterior (TLIF/PLIF) or direct anterior (ALIF) approaches without comparative data for LLIF/OLIF. 2. Articles that reported general fusion rates but lacked granular data on anatomical risks or specific neurovascular complication profiles. 3. Case reports, technical notes with n < 5 patients, conference abstracts, and non-peer-reviewed white papers. 4. Preliminary reports where the data were later incorporated into a larger, more comprehensive multicenter study are already included in the review.

Lateral lumbar/transpsoas interbody fusion (LLIF/LTIF): anatomical corridor and associated risks

Surgical Approach: The Direct Lateral Retroperitoneal Transpsoas Corridor

Lateral lumbar interbody fusion (LLIF) is a minimally invasive lateral transpsoas approach to lumbar fusion, with several commercially branded systems commonly referred to as extreme lateral interbody fusion (XLIF) or direct lateral interbody fusion (DLIF). [14]. This surgical technique is utilized for the treatment of various degenerative spinal conditions, including disk herniation, degenerative disc disease, and spinal stenosis [15]. It is one of several surgical approaches for lumbar interbody fusion, which also include anterior (ALIF), oblique (OLIF), transforaminal (TLIF), and posterior (PLIF) methods [14]. The fundamental rationale for the development of LLIF was to address and mitigate the significant complications associated with traditional open spinal fusion surgeries, particularly the extensive disruption of paraspinal muscles, prolonged nerve root retraction, and substantial blood loss [14].

The LLIF procedure establishes a unique surgical corridor that circumvents the posterior and anterior musculature and structures [16]. The patient is positioned on their side (lateral decubitus), and the surgeon makes a small incision in the flank. The approach is retroperitoneal, meaning the surgeon gently navigates the space behind the peritoneum, which contains the abdominal organs, and moves these organs and major vessels anteriorly. This part of the approach spares the visceral organs and provides a direct line of access to the lateral aspect of the lumbar spine [14].

The defining characteristic of the LLIF corridor is the final passage through the psoas major muscle to reach the intervertebral disk [17]. This transpsoas trajectory allows for the insertion of a large interbody graft, which has several clinical advantages. A wider cage footprint can be achieved, resting on the dense apophyseal ring of the vertebral endplates, which enhances stability [18]. Furthermore, the restoration of disc height by the inserted implant can lead to indirect decompression of neural elements and significant lordosis correction. While the lateral approach avoids many of the risks of posterior and anterior procedures, such as dural tears and sympathetic plexus injury, it introduces its own set of complications related to the structures that must be traversed to reach the target disk [14]. This distinct risk profile, particularly concerning neuromuscular injury, is what differentiates it from other lateral approaches like OLIF, which access the disk from a corridor anterior to the psoas [14]. The approach thus represents a calculated surgical trade-off, where the benefits of a minimally invasive technique are weighed against the unique anatomical risks inherent to the transpsoas corridor. 

Relevant Anatomy: Psoas, Lumbar Plexus, and Major Vasculature

A thorough understanding of the intricate anatomy within and adjacent to the transpsoas surgical corridor is essential for safe and effective LLIF. The major structures of concern include the psoas muscle itself, the lumbar plexus embedded within it, and the great retroperitoneal vessels [19]. A nuanced appreciation for the anatomical variability of these structures is paramount, as their position can differ significantly by vertebral level and patient laterality, directly influencing the risk of iatrogenic injury.

The psoas major muscle is a large, key muscle of the posterior abdominal wall that originates from the transverse processes and lateral surfaces of the lumbar vertebrae. A crucial anatomical detail for the LLIF approach is that the lumbar plexus, a complex network of nerves, is formed by the anterior rami of spinal nerves L1-L4 and is intimately embedded within the posterior aspect of the psoas muscle. This close anatomical relationship means that any surgical path through the psoas muscle, particularly for LLIF, places the nerves of the lumbar plexus directly in the surgical field and makes them vulnerable to injury from retraction or direct trauma.

A seminal study investigated the position of these neurovascular structures relative to the transpsoas approach using MRI [3]. This research established a key relationship, demonstrating that the location of the lumbar plexus can be inferred from the position of the psoas muscle [3]. The study found a predictable pattern of risk, with the percentage of patients having neurovascular structures at risk increasing significantly at lower lumbar levels [3].

The abdominal aorta and the inferior vena cava (IVC) are the two largest blood vessels in the abdomen, lying retroperitoneally along the anterior aspect of the vertebral column. Their anatomical position is a critical determinant of surgical risk. The abdominal aorta typically lies to the left of the midline, while the IVC is positioned to the right. This anatomical asymmetry has profound implications for the LLIF approach, as a right-sided approach requires navigating a corridor in close proximity to the IVC and the right common iliac vein, which lies to its posterior aspect [20]. The IVC is a thin-walled vessel, making it particularly susceptible to injury.

Kepler and colleagues' study provided a demonstration that the position of these great vessels, particularly the IVC, is a significant factor contributing to the overall risk profile of LLIF [3]. The study's data revealed that the percentage of patients with neurovascular structures at risk was substantially higher for a right-sided approach compared to a left-sided approach, largely due to the more posterior position of the right-sided vasculature [3]. This difference in risk is most pronounced at the L4-5 level, where the iliac vessels are located, and the IVC terminates. The anatomical reality is that a right-sided, lower lumbar approach is a much more challenging and higher-risk endeavor, requiring a heightened level of surgical caution and meticulous pre-operative planning. 

Neurovascular Risks and Mechanisms of Injury

The unique anatomical corridor of the LLIF procedure, while offering significant benefits, presents a distinct set of neurovascular risks. These risks are not uniform but are heavily influenced by the specific vertebral level and the side of the surgical approach. A comprehensive understanding of the mechanisms of injury and the specific structures at risk is essential for effective surgical planning and mitigation.

The primary mechanisms of nerve injury during the transpsoas approach are related to the retraction of the psoas muscle, which can result in nerve compression, stretch, and compromised vascular perfusion. The nerves of the lumbar plexus, being embedded within the psoas, are susceptible to these forces [5]. The risk of a lumbar plexus injury has been correlated with factors such as longer retraction times, multi-level approaches, and patient positioning. However, more recent studies and advanced neuromonitoring data have refined this understanding. These data suggest that the injury is not solely a function of retraction duration but rather the quality and pressure of the retraction. A brief period of intense compression may be more damaging than a longer period of low-pressure retraction. This finding has led to a re-evaluation of surgical protocols, shifting the focus from simple time limits to a more nuanced, function-based approach guided by real-time monitoring.

The most significant nerves at risk during the transpsoas approach are branches of the lumbar plexus, particularly the femoral nerve. As the largest branch, the femoral nerve innervates the primary hip flexors and knee extensors; high-grade injury can be debilitating, resulting in persistent anterior thigh pain, numbness, and motor deficits such as hip flexor weakness. The genitofemoral nerve, coursing through the psoas, is also vulnerable, and its injury may cause sensory loss in the groin and anterior thigh. Although the obturator nerve is less frequently affected during LLIF, its proximity within the psoas makes it a potential concern, with injury leading to adductor weakness. Collectively, these neuropathic complications, most commonly anterior thigh pain and numbness, remain prevalent despite intraoperative neuromonitoring.

Vascular injuries are a rare but potentially devastating complication of LLIF [18]. A critical finding from anatomical studies is the disproportionately high risk associated with right-sided approaches, particularly at the L4-5 level [20]. The reason for this heightened risk is the anatomical positioning of the inferior vena cava (IVC) and the right common iliac vein, which are located more posteriorly relative to the spine compared to the aorta on the left [20].

The data from the study by Kepler et al. clearly illustrate this disparity [3]. As shown in Table 1, the percentage of patients with a neurovascular structure at risk is dramatically higher for right-sided approaches, culminating in a 44.2% risk at L4-5 [3]. While most vascular injuries in LLIF are minor, such as segmental vessel lacerations, injuries to major vessels like the IVC can be catastrophic [18]. This data provides a powerful and specific rationale for why a surgeon's choice of laterality and level must be informed by a meticulous pre-operative assessment of the patient's individual anatomy. As summarized in Table 1, the prevalence of neurovascular structures at risk increases at lower lumbar levels and is consistently higher for right-sided LLIF approaches, with the greatest disparity observed at L4-5.

**Table 1:** Prevalence of Neurovascular Structures at Risk by Spinal Level and Surgical Laterality in LLIF Approaches Data derived from Kepler et al. [3] LLIF: lateral lumbar interbody fusion

Spinal Level	% of Patients with Structures at Risk (Left-Sided Approach)	% of Patients with Structures at Risk (Right-Sided Approach)
L1-2	2.30%	7.00%
L2-3	7.00%	7.00%
L3-4	4.70%	9.30%
L4-5	20.90%	44.20%

Strategies for Mitigation: Preoperative Planning and Intraoperative Vigilance

Given the predictable, level- and side-specific risks of the transpsoas approach, the modern LLIF procedure relies heavily on a multi-pronged strategy of risk mitigation. This strategy begins long before the first incision and extends throughout the entire surgical procedure.

Meticulous preoperative imaging is the cornerstone of safe LLIF surgery. While imaging, such as MRI, is standard for identifying spinal pathology, its role in LLIF extends to a detailed assessment of the surgical corridor. Surgeons must carefully evaluate the location of the major retroperitoneal vasculature, the lumbar plexus, and the genitofemoral nerve relative to the psoas muscle and the planned surgical trajectory. The purpose of this detailed mapping is to identify individual anatomical variations that could place a patient at heightened risk [19]. For example, the knowledge that a patient's IVC is positioned more posteriorly at the L4-5 level on the right side can influence the surgeon's choice of approach, leading them to either select a different laterality, a different fusion technique, or to proceed with extreme caution and heightened vigilance [19]. This preoperative planning transforms the approach from a standardized procedure into a patient-specific, anatomically-guided intervention.

Intraoperative neuromonitoring (IONM) is considered a standard and essential tool for mitigating neural injury during LLIF. The technique involves real-time electrophysiological surveillance using a combination of electromyography (EMG), somatosensory evoked potentials (SSEPs), and motor evoked potentials (MEPs) [16]. The use of IONM has been shown to reduce the risk of postoperative deficits associated with navigating through the psoas muscle. A key development in this field is the use of femoral nerve evoked potentials (FNEPs). Studies have demonstrated that FNEP alerts can provide a timely indication of "hyperacute femoral nerve conduction failure". When these alerts are triggered, surgeons can employ immediate countermeasures, such as adjusting or removing the retractor. This reactive adjustment is crucial, as studies have found that nerve function often recovers to baseline, and postoperative deficits are avoided when prompt action is taken. This ability to detect nerve impairment before it becomes a permanent injury is a profound shift in surgical safety, moving beyond the outdated notion that limiting retraction time is the primary determinant of risk.

The LLIF procedure itself is performed with a focus on meticulous technique to minimize tissue and neural disruption [17]. The surgical corridor is created using a series of progressively larger tubular dilatators rather than a single, large incision [17]. This process relies on blunt dissection to carefully separate muscle fibers and displace the lumbar plexus nerves within the psoas, reducing the risk of direct trauma or laceration. Real-time fluoroscopy (X-ray) is used to guide the instruments to the correct vertebral level, while IONM provides continuous surveillance for nerve integrity [17]. For vascular risks, the approach’s reliance on blunt, retroperitoneal dissection and the absence of a large open incision significantly reduces the risk of injury to the great vessels [14]. In the rare event of a vascular injury, a mini-open technique can provide direct visualization and allow for immediate repair, often without long-term sequelae. This highlights the importance of not only avoiding complications but also having a clear plan for their immediate management.

Oblique lumbar interbody fusion (OLIF): anatomical corridor, associated risks, and mitigation strategies

The Surgical Approach: The Oblique Pre-Psoas or Anterior-to-Psoas Corridor

Oblique Lumbar Interbody Fusion (OLIF) is a minimally invasive surgical technique that has gained significant traction as an alternative to traditional spinal fusion methods, including anterior lumbar interbody fusion (ALIF), posterior lumbar interbody fusion (PLIF), transforaminal lumbar interbody fusion (TLIF), and direct lateral lumbar interbody fusion (LLIF). Its development traces back to Mayer’s retroperitoneal antepsoas technique, with the procedure formally named and detailed in subsequent large clinical series [21, 22]. Modern refinements were pioneered by surgeons like Richard Hynes, who applied a tubular retractor system and expanded the approach to include the challenging L5-S1 disc space, leading to the contemporary OLIF procedure [23].

The growing popularity of OLIF is attributed to several theoretical advantages that address the limitations of other techniques. The minimally invasive nature of the procedure is associated with reduced blood loss, improved postoperative pain management, and a potentially faster recovery due to minimized tissue disruption [24]. A key procedural benefit is the ability to address multiple lumbar levels, from L2 to S1, from a single patient position, which streamlines the surgical process and enhances efficiency. Unlike anterior lumbar interbody fusion (ALIF), which often requires the assistance of a vascular surgeon and carries risks of major visceral and vascular injuries, or direct lateral lumbar interbody fusion (LLIF), which requires traversing the psoas muscle and is limited at the L5-S1 level by the iliac crest, OLIF provides a versatile solution for a wide range of degenerative and deformity spinal conditions. The OLIF approach capitalizes on the oblique corridor, passing anterior to the psoas muscle, thereby significantly reducing the risk of psoas-related nerve injury commonly associated with LLIF, and minimizing vascular risk compared to ALIF.

The essence of the OLIF approach lies in its unique surgical trajectory, which is defined as a mini-open, muscle-sparing, and nerve-avoiding procedure [22]. The technique is performed in the lateral decubitus position, typically on the right side for a left-sided approach. The surgeon makes an oblique incision, often located in the left lower quadrant of the abdomen, approximately two fingerbreadths anterior to the anterior iliac crest [23]. This incision allows for a muscle-splitting approach through the external oblique, internal oblique, and transversus abdominis muscles, leading to the retroperitoneal space and allowing direct access to the spine anterior to the psoas muscle [22, 23].

Within the retroperitoneal space, blunt dissection is performed to sweep the peritoneum and its contents anteriorly, exposing the lumbar spine without entering the peritoneal cavity. The surgical corridor itself is an avascular plane located between the medial border of the left psoas muscle and the lateral aspect of the great vessels, namely the abdominal aorta and inferior vena cava. By utilizing this natural, pre-existing anatomical space, the OLIF procedure is fundamentally designed to avoid direct dissection or splitting of the psoas muscle, a core technical distinction that defines its risk profile and distinguishes it from the transpsoas approach of LLIF. This approach is often referred to as "anterior to psoas" (ATP), reflecting its foundational principle of bypassing the muscle and its contents [5].

Relevant Anatomy of the Operative Window

The successful execution of an OLIF procedure is predicated on a profound understanding of the retroperitoneal anatomy. The retroperitoneal space is the primary operative field, bounded anteriorly by the peritoneum and posteriorly by the vertebral column and psoas muscles. It contains the great vessels, kidneys, pancreas, and portions of the digestive tract. The OLIF approach respects the integrity of the peritoneum, sweeping it anteriorly with blunt dissection to access the lumbar spine.

The psoas muscle forms the lateral boundary of the surgical corridor. The lumbar plexus, which includes critical nerves such as the genitofemoral, femoral, and obturator nerves, is located within the substance of the psoas muscle or deep to it [1]. The design of the OLIF technique aims to remain anterior to the psoas, thereby theoretically preserving the muscle fibers and the nerves contained within. This strategic avoidance is a direct response to the high rates of lumbar plexus injury observed in transpsoas approaches like LLIF. However, these structures remain at risk from compression or prolonged retraction, which can still lead to neurological symptoms.

The great vessels, including the abdominal aorta and inferior vena cava (IVC), define the medial boundary of the corridor. The abdominal aorta is consistently positioned on the left anterolateral side of the lumbar spine, while the IVC is located on the right anterolateral side [23]. The standard left-sided OLIF approach is chosen to utilize the space between the aorta and the left psoas muscle. This is a deliberate choice to avoid the thin-walled, less elastic IVC, which is more susceptible to intraoperative injury [23]. At the L5-S1 level, however, the anatomy becomes more complex due to the bifurcation of the great vessels and the obstruction of the iliac crest. Here, different approaches may be necessary, including left intra-bifurcation, left pre-psoas, or a right-sided pre-psoas approach [26]. The position of the left common iliac vein (CIV) is particularly critical at L5-S1, as injury risk increases when it is located over the medial two-thirds of the disc space [25].

The lumbar sympathetic chain (LSC) is a critical anatomical structure that runs along the anterolateral aspect of the lumbar vertebral bodies, often situated at the medial border of the psoas muscle. Due to its proximity, it is a key structure in the surgical field and is susceptible to iatrogenic injury during the procedure [10].

Neurovascular Risks and Complication Analysis

Despite its design to minimize neurological complications, the OLIF procedure is not without risk to the nervous system. The most commonly reported neurological complications are related to the lumbar sympathetic chain and the lumbar plexus.

Lumbar sympathetic chain injury (LSCI) is a recognized, yet frequently underestimated, complication of OLIF, with reported incidence rates varying widely from 0.5% to 7.3% across different studies, likely due to a lack of standardized diagnostic criteria [26]. The mechanism of injury is typically iatrogenic, involving compression or traction on the chain during instrument manipulation, particularly when it is visible within the surgical field. Clinical manifestations of LSCI are caused by a transient or permanent disruption of sympathetic function. They include increased skin temperature, anhidrosis (inability to sweat), swelling, skin discoloration, and dysesthesia of the affected limb. The implication is that even though the OLIF approach aims to avoid nerves, careless or extended tissue retraction can negate this advantage, leading to avoidable complications. This points to a direct, actionable recommendation for surgeons: minimize the duration and force of tissue retraction to preserve neural integrity.

Although the OLIF approach is designed to avoid the lumbar plexus, it remains susceptible to injury through mechanisms of prolonged compression, excessive traction, or inadvertent stripping of the psoas muscle [26]. A retrospective analysis of 155 patients reported that thigh symptoms were the most common approach-related complication, with an incidence of ipsilateral transient psoas paresis and transient quadriceps weakness in 13.5% [27]. While these symptoms are often transient and tend to decrease over time, they can be persistent, underscoring the importance of meticulous technique [28].

While the OLIF procedure is designed to reduce the risk of vascular injury compared to ALIF, vascular complications remain the most threatening risk associated with the procedure [2]. The risk of vascular injury is known to increase at the L5-S1 level, with one study reporting a rate of 2.5% when this level was included [26]. The primary risk at this level is injury to the left common iliac vein, which is particularly vulnerable when it overlies the medial two-thirds of the disc [26].

The risk of vascular injury is not confined to the direct surgical corridor. A cadaveric study identified an underappreciated risk of injury to the contralateral lumbar segmental arteries during discectomy and cage insertion [18]. These vessels lie outside the direct field of view and are at risk due to the "blind" nature of instrument manipulation [18]. This finding reveals a hidden, yet critical, risk that requires heightened intraoperative awareness [18]. The study also established a strong correlation between the positions of the left and right segmental arteries at the L4 and L5 levels, suggesting that the intraoperative location of a high-risk artery on the approach side can serve as a predictive indicator for a similar risk on the contralateral side [18]. This predictive anatomical relationship transforms the awareness of a risk into an actionable piece of information, guiding surgeons to proceed with greater caution during these blind maneuvers to avoid complications such as a postoperative psoas hematoma [18].

Comparative analysis of risks and anatomical considerations

Plexus Proximity

The fundamental difference between lateral lumbar interbody fusion (LLIF) and oblique lumbar interbody fusion (OLIF) lies in the surgical corridor relative to the psoas muscle and the lumbar plexus [23]. The LLIF procedure is a "transpsoas" approach, meaning the surgeon's instruments pass directly through the psoas muscle to access the disc space. The lumbar plexus, which includes the femoral and genitofemoral nerves, is embedded within the posterior portion of the psoas [23].

Although the OLIF approach is designed to avoid the lumbar plexus, it remains susceptible to injury through mechanisms of prolonged compression, excessive traction, or inadvertent stripping of the psoas muscles [26]. These symptoms are often transient and tend to decrease over time; they can be persistent, underscoring the importance of meticulous technique [26].

Vascular Risks

While the LLIF approach is designed to navigate around major vessels and visceral organs, it is not without vascular risk. A right-sided LLIF approach is particularly hazardous due to the more posterior position of the inferior vena cava (IVC) and right iliac vein relative to the spine [29].

The OLIF approach, on the other hand, operates in the narrow corridor between the psoas muscle and the great vessels, including the aorta and IVC. This anatomical proximity makes the procedure more susceptible to vascular injuries, which are considered the most serious complication of OLIF [2]. 

Overall Neurological Complication Rates

Systematic reviews and meta-analyses comparing OLIF and LLIF in the L1-L5 region demonstrate a clear inverse relationship between the primary risks of these two procedures [7]. The direct transpsoas approach of LLIF is associated with a higher incidence of neurological complications, such as transient neuropraxia and sensory deficits, compared to the pre-psoas OLIF approach. Conversely, the OLIF approach presents a relatively higher risk for vascular injury. These distinct risk profiles are directly linked to the specific anatomical corridors utilized by each procedure.

Impact of Anatomical Variations

Patient-specific anatomical factors, such as variations in vascular and neural anatomy, are crucial for determining the safest surgical approach. 

Vascular Variations: In OLIF, a rare but significant congenital anomaly, such as a left-sided IVC, which occurs in 0.2% to 0.5% of the population, can make the standard left-sided approach unsafe [30]. This anomaly drastically downsizes the surgical corridor and necessitates an alternative, such as a right-sided approach, to avoid significant vascular damage.

**Figure 2:**
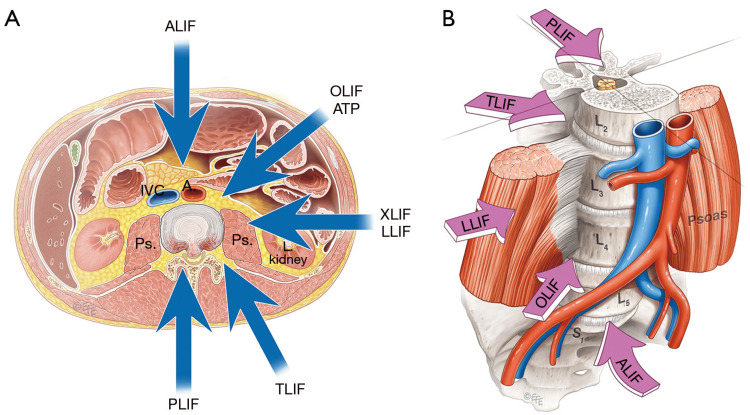
Overview of Lumbar Interbody Fusion Surgical Approaches (A) Schematic representation of the principal access routes to the lumbar spine, including anterior (ALIF), lateral/extreme lateral (LLIF/XLIF), oblique/anterior-to-psoas (OLIF/ATP), transforaminal/minimally invasive (TLIF/MI-TLIF), and posterior (PLIF) lumbar interbody fusion approaches. (B) Illustration highlighting how the relative position of the psoas muscle and anterior vascular structures influences approach selection across lumbar levels. Reproduced from Mobbs et al. [32] under the terms of the Creative Commons Attribution–NonCommercial–NoDerivatives 4.0 (CC BY-NC-ND 4.0) license; permission for reuse is granted by the original publisher under this license. Ps.: psoas muscle; IVC: inferior vena cava; A: artery; L2-L5: lumbar vertebrae 2-5; S1: sacral vertebra 1

Lumbar Plexus Variations: Variations in the lumbar plexus are common, with prevalence rates ranging from 8.8% to 47.1% for individual nerves [31]. Because LLIF operates directly within the psoas muscle, where the plexus resides, it is highly susceptible to complications arising from these variations [1]. A study on the LLIF approach found a relationship between the psoas muscle's position and the lumbar plexus, suggesting that the psoas's location can serve as a proxy for identifying patients at risk of injury, especially at the L4-5 level [19]. To contextualize the anatomical considerations underlying approach selection, Figure 2 illustrates the principal lumbar interbody fusion corridors and their spatial relationships to the psoas muscle and anterior vascular structures, as referenced in Mobbs et al. [32].

Advantages and Disadvantages from an Anatomical Risk Perspective

The comparative analysis reveals a clear anatomical risk-benefit trade-off between LLIF and OLIF. The choice of which procedure to perform should be guided by a thorough evaluation of the patient’s specific anatomy to mitigate the most significant risks. Table 2 summarizes the key anatomical advantages and disadvantages of LLIF and OLIF, highlighting the distinct risk profiles associated with each surgical corridor.

**Table 2:** Anatomical Risk–Benefit Comparison of LLIF and OLIF Surgical Approaches OLIF: oblique lumbar interbody fusion; LLIF: lateral lumbar interbody fusion; IVC: inferior vena cava

Surgical Approach	Anatomical Advantages (Risk Reduction)	Anatomical Disadvantages (Increased Risk)
LLIF (Transpsoas)	Lower risk of major vascular injury compared to OLIF. The approach spares the great vessels and abdominal viscera by traversing the psoas muscle.	Higher risk of neurological injury, specifically to the lumbar plexus and femoral nerve. The approach's trajectory can be compromised by the variable position of the IVC, especially with right-sided approaches at L4-5.20
OLIF (Pre-psoas)	Significantly lower risk of injury to the lumbar plexus, as the approach avoids direct psoas muscle dissection. Associated with a lower incidence of hip flexion weakness and thigh numbness.	Higher risk of vascular injury to the great vessels (aorta and IVC) due to operating in close proximity to them. Also carries a notable risk of sympathetic chain injury from prolonged retraction.

## Conclusions

The literature demonstrates that both lateral lumbar interbody fusion (LLIF) and oblique lumbar interbody fusion (OLIF) are effective minimally invasive techniques with distinct anatomical corridors and corresponding neurovascular risk profiles. The transpsoas approach of LLIF primarily exposes the lumbar plexus to neural injury. In contrast, the pre-psoas corridor used in OLIF reduces neurological risk at the expense of increased proximity to major vascular structures. These inherent differences highlight the need for a careful approach selection tailored to individual patient anatomy.

Optimal outcomes depend on meticulous preoperative anatomical assessment and sustained intraoperative vigilance. Recognition of patient-specific variations, including vascular anomalies, is essential for safe surgical planning, while multimodal neuromonitoring and judicious retraction strategies remain critical for risk mitigation. Future research should prioritize standardized outcome reporting, improved imaging for surgical planning, and the evaluation of emerging technologies, such as navigation and robotics, to further enhance procedural safety.
